# *Berkeleyomyces rouxiae*—A Pathogen Causing the Black Root Rot of Tobacco

**DOI:** 10.3390/pathogens13121120

**Published:** 2024-12-18

**Authors:** Grażyna Korbecka-Glinka, Anna Trojak-Goluch, Diana Czarnecka

**Affiliations:** Department of Biotechnology and Plant Breeding, Institute of Soil Science and Plant Cultivation—State Research Institute, 24-100 Puławy, Poland; anngol@iung.pulawy.pl (A.T.-G.); diana.czarnecka@iung.pulawy.pl (D.C.)

**Keywords:** *Nicotiana tabacum*, *Berkeleyomyces rouxiae*, *Thielaviopsis basicola*, *Chalara elegans*, cryptic species identification, pathogenicity

## Abstract

Black root rot is a dangerous disease affecting many crops. It is caused by pathogens formerly known as *Thielaviopsis basicola* and then reclassified as two cryptic species, *Berkeleyomyces basicola* and *B. rouxiae*. The aim of this study was to perform species identification, morphological characterization, and pathogenicity tests for fungal isolates obtained from tobacco roots with black root rot symptoms in Poland. DNA sequences of the three regions (ITS, *ACT*, *MCM7*) were highly similar to the sequences of *B. rouxiae* deposited in the NCBI database. Phylogenetic analysis confirmed the assignment of the obtained isolates to this species. The cultures of four representative isolates (namely OT2, OT3, WPT7, WPT8) showed a similar structure and gray/brown color of the mycelium, although their growth rate varied from 3.8 to 5.1 mm/day depending on the isolate. The sizes of the endoconidia and chlamydospores showed a considerable variation, although they fit within ranges previously described for *B. rouxiae*. Pathogenicity tests performed on young tobacco plants grown in the inoculated peat substrate revealed differences among the four isolates. WPT7 demonstrated the lowest level of aggressiveness for tobacco. In contrast, the remaining three isolates caused severe disease symptoms and significantly reduced shoot and root dry weights of the susceptible cultivar Virginia Joyner. A parallel pathogenicity test performed on cultivar VRG 10TL confirmed the effectiveness of black root rot resistance derived from *Nicotiana debneyi*.

## 1. Introduction

**Figure 1:**
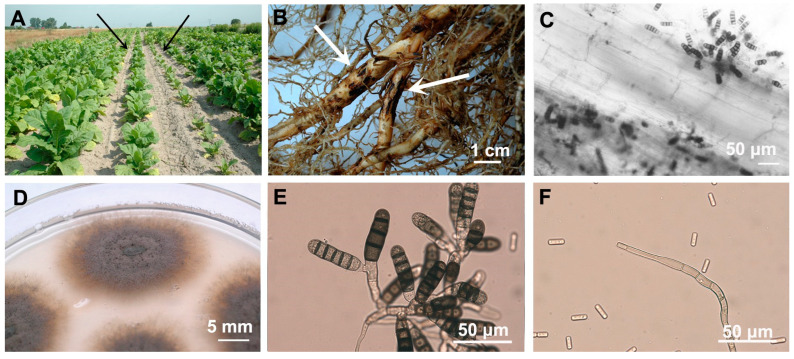
The symptoms of black root rot on tobacco and the morphology of its causative agent. (**A**) Tobacco field with cultivars differing with their susceptibility to black root rot. Plants in the middle rows (indicated with black arrows) show symptoms of the disease such as yellowing and retarded growth. (**B**) Dark lesions (indicated with white arrows) on the surface of the tobacco roots caused by *B. rouxiae* in the field conditions (bar = 1 cm). (**C**) Aleuriospores and hyphae of *B. rouxiae* produced in/on tobacco diseased roots (bar = 50 µm). (**D**) Colony morphology of *B. rouxiae* after 8 days of growth on PDA at 25 °C in darkness (bar = 5 mm). (**E**) Aleuriospores of *B. rouxiae* isolate OT3 produced on PDA (bar = 50 µm). (**F**) Phialide and cylindrical endoconidia of *B. rouxiae* isolate OT3 (bar = 50 µm).

Black root rot is a disease affecting over 170 plant species including important ornamental and field crops, such as cotton, carrot, lettuce, peanut, and tobacco [1]. It has been reported in most continents and has been the cause of major epidemics on the fields of cotton or tobacco [2,3]. The disease is caused by a soil-borne fungus that colonizes the roots of plants and, after a short biotrophic phase, it switches to a necrotrophic lifestyle, causing the blackening and gradual decay of the root system (Figure 1B,C). Impaired supply of water and nutrients to the shoots leads to the discoloration of leaves, a loss of turgor, and the stunted growth of plants [2] (Figure 1A). Black root rot affects tobacco, mostly in regions with cooler climates such as Canada, a few states of the USA (e.g., Kentucky), and Europe, where severe outbreaks have been noted in Poland, Germany, and Italy [4]. Losses of the crop in the case of susceptible cultivars can reach 10–50% [5,6].

A causative agent of black root rot until recently was known as *Thielaviopsis basicola* (Berk. and Broome) Ferraris [syn. *Chalara elegans* Nag Raj and W.B. Kendr.]. Taxonomic revision of the fungi from the Ceratocystidaceae family revealed that *T. basicola* may form a lineage separate from the *Thielaviopsis* genus [7]. A subsequent study based on sequencing six different gene regions not only confirmed these results, but also distinguished within this lineage two sister species, which were named *Berkeleyomyces basicola* and *B. rouxiae* [8]. These species are considered to be cryptic, as it is not possible to distinguish them based on the morphological traits without DNA sequencing and phylogenetic analysis.

Over the last few years, the taxonomic identity of fungi, previously known as *T. basciola,* has been verified based on DNA sequence data for isolates from different hosts. *B. basicola* was isolated from carrot and lisianthus [9,10]. On the other hand, *B. rouxiae* was discovered on cotton, lettuce, peanut, hemp, and faba bean [11,12,13,14,15]. Nevertheless, given the global threat that black root rot poses to numerous significant crops, it is essential to obtain further data on the DNA-based identification of its causal agents. These findings will contribute to the currently limited knowledge on the geographic distribution and host range of both *Berkeleyomyces* species [1]. The objective of this research is to characterize fungal isolates obtained from tobacco plants with black root rot symptoms in Poland and to verify their taxonomic identity by sequencing informative DNA regions.

## 2. Materials and Methods

### 2.1. Obtaining Fungal Isolates from Tobacco Roots

The roots of eight tobacco plants of the Virginia Joyner cultivar displaying mild symptoms of black root rot were collected in the fields at two locations: Osiny (51°28′ N, 22°03′ E) and Wola Przybysławska (51°24′ N, 22°19′ E), situated in Lubelskie province (southeastern Poland). For comparison, we also harvested roots from the greenhouse tobacco plants that have been inoculated with the *T. basicola* isolate originating from the collection of the Institute of Soil Science and Plant Cultivation—State Research Institute. This isolate was previously used in inoculation experiments performed to select tobacco breeding lines resistant to black root rot [16] and then maintained in the authors’ collection of fungal isolates.

Fungal isolates were obtained from the collected roots using a modified carrot bait method [17]. Peeled carrots were surface disinfected by means of sodium hypochlorite solution (with approx. 1.5% active chlorine) for two minutes, rinsed twice in sterile water, and dried on filter tissue paper under sterile airflow in a class II microbiological safety cabinet (NordicSafe^®^, Esco, Singapore). Disinfected carrots were cut into 2–3 mm thick discs, which were then placed on wet filter paper in Petri dishes (6 discs per Petri dish). The tobacco roots of each of the eight plants were washed under running tap water, and then symptomatic root fragments (with a cracked and dark-colored surface) were cut into small pieces (2–10 mm long) and placed separately on five carrot discs in each Petri dish. The sixth disc in each Petri dish was kept without explants, as a control. All carrot discs were moistened with one drop of sterile water. Then, the Petri dishes were sealed with parafilm and incubated in the dark at room temperature. After 2–10 days, samples of mycelium grown around the explants were examined under a light microscope in search of structures characteristic of *Berkeleyomyces* (e.g., phialides, endoconidia, chlamydospores). No fungal growth was observed on the control carrot discs without explants. Samples of mycelium which were confirmed to contain the above-mentioned structures were transferred into Petri dishes containing Potato Dextrose Agar (PDA; Difco, Sparks, MD, USA) amended with tetracycline hydrochloride (2.5 mg/L). The obtained *Berkeleyomyces* cultures were first purified by transfers of small pieces of mycelium to fresh PDA medium. Then, monosporic isolates were obtained from isolating and transferring single germinating endoconidia to fresh PDA medium. The morphological structures of the obtained isolates were observed and documented using an Eclipse 80i microscope (Nikon, Tokyo, Japan).

### 2.2. Molecular Species Identification of the Obtained Isolates

Single-spore isolates subjected to molecular identification included four isolates from different plants collected in each sampling location (Osiny and Wola Przybysławska) and two isolates from the greenhouse plants. For comparative purposes, we also included cultures of the following *T. basicola* isolates collected in Poland and maintained by the Bank of Pathogens of the Institute of Plant Protection—National Research Institute (IPP-NRI; Poznań, Poland):

- isolate IPP-590 obtained from wheat (*Triticum vulgare* Vill.);
- isolate IPP-96 obtained from poinsettia (*Euphorbia pulcherrima* Willd. ex Klotzsch);
- isolate IPP-2133 obtained from carrot (*Daucus carota* L.).

Cultures of all the selected isolates were subjected to DNA extraction using a modified CTAB method [18]. The extraction buffer consisted of 3% *w*/*v* CTAB, 100 mM Tris-base, 20 mM EDTA, 1.4 M NaCl, pH = 8. The concentration and purity of the obtained DNA extracts were assessed by means of NanoDrop2000 (Thermo Scientific, Wilmington, DE, USA). Then, all the isolates were subjected to the amplification and sequencing of the three DNA regions:

- internal transcribed spacer region (ITS) using primers ITS1-F and ITS4 [19];
- minichromosome maintenance complex component 7 (*MCM7*) using primers Cer-MCM7F and Cer-MCM7R [7];
- actin (*ACT*) using primers CeractF1 and CeractR2 [8].

The polymerase chain reaction (PCR) amplification for all three regions was performed in a volume of 20 μL containing 10 μL of Platinum II Hot Start PCR 2× Master Mix (Invitrogen, Vilnus, Lithuania), 0.2 μM of each of the two primers, and 50 ng of DNA. PCR was performed by means of a C1000 thermal cycler (Bio-Rad, Singapore) using a thermal program consisting of initial denaturation at 94 °C for 2 min and 35 cycles of denaturation at 94 °C for 15 s, annealing (at 55 °C for *MCM7* and at 60 °C for two other regions) for 15 s, and extension at 68 °C for 15 s. Subsequently, the PCR products were treated with ExoSAP-IT reagent according to the manufacturer’s protocol (Applied Biosystems, Vilnus, Lithuania) and subjected to sequencing. Cycle sequencing was performed using Big Dye Terminator v3.1 chemistry (Applied Biosystems, Vilnus, Lithuania) and Veriti thermal cycler (Applied Biosystems, Singapore). Then, sequencing products were purified using ethanol/EDTA precipitation and separated on a 3500 Genetic Analyzer (Applied Biosystems, Ibaraki, Japan). The sequences were initially reviewed and trimmed using Sequencing Analysis software v.6.0 (Applied Biosystems, Foster City, CA, USA). 

Forward and reverse sequences for each genomic region and each isolate were assembled into continuous sequences using MEGA v.11 [20]. Then, the sequences of all tobacco isolates were aligned using Clustal W to visualize polymorphic sites. They were also subjected to a search of highly similar sequences in the database of the National Center for Biotechnology Information (NCBI) using the Basic Local Alignment Search Tool (BLAST; http://blast.ncbi.nlm.nih.gov accessed on 14 October 2024). 

For further research, we selected four representative isolates obtained from different tobacco plants: two from Osiny (isolate names: OT2 and OT3) and two from Wola Przybysławska (isolate names: WPT7 and WPT8). Cultures of these isolates were deposited in the CBS Collection of the Westerdijk Fungal Biodiversity Institute (The Netherlands) under the following accession nos.: CBS152705–CBS152708. DNA sequences of these four isolates and the three isolates obtained from the Bank of Pathogens IPP-NRI were deposited in the NCBI.

In order to construct a phylogenetic tree, additional sequences of *B. basicola*, *B. rouxiae* and several related species were downloaded from the NCBI database (Table S1). Sequences of the type specimens were selected, if available. Since there were very few *ACT* sequences available for the related species in NCBI database, the first phylogenetic analysis, including 40 accessions, was based on the concatenated sequences of ITS and *MCM7*. Then, all three regions were used to construct a phylogenetic tree for 27 accessions belonging to *Berkeleyomyces* and *Ceratocysits* genera. The sequences were concatenated and aligned using Clustal W in MEGA v.11 [20]. Then, a maximum likelihood method and Kimura 2-parameter model were used in the same software to construct a phylogenetic tree. A phylogeny test was performed using 1000 bootstrap replicates.

### 2.3. Detailed Morphological Characterization of Representative Isolates

Discs of 6 mm diameter were cut from cultures of the four selected isolates (OT2, OT3, WPT7, WPT8) and transplanted into Petri dishes with fresh PDA medium (each isolate in five replicates). After incubation at 25 °C, in the dark for 11 days, the diameters of the cultures were measured and the growth rate (GR) was calculated according to the formula GR = (d − 6)/11, where d is the culture diameter at day 11. 

For every isolate, the length and width of 100 chlamydospore segments were measured and their number per aleuriospore was counted. In addition, the length and width of 100 endoconidia and 100 conidiogenous cells (phialides) were measured. As phialides had a tapering shape, their width was measured twice, in their widest and narrowest point (near base and at tip). All microscopic observations and measurements were performed by means of Eclipse 80i microscope with NIS-Elements AR 3.2 software (Nikon, Tokyo, Japan).

### 2.4. Testing Pathogenicity of the Representative Isolates

Two tobacco cultivars differing in their susceptibility to black root rot were selected for testing the pathogenicity of the four representative fungal isolates. The cultivar Virginia Joyner of United States origin is very susceptible to *T. basicola* and, therefore, is often used as a susceptible standard in pathogenicity tests [21]. The second cultivar, VRG 10TL, is widely grown in Poland and it is resistant to black root rot because it carries a resistance originating from *Nicotiana debneyi* [22,23]. The seeds of both cultivars were surface-disinfected in 10% hydrogen peroxide for 20 min and then thoroughly rinsed with water and sown into the peat substrate. After two weeks, seedlings were transplanted into a cavity tobacco seedling tray with 160 individual cells. Approximately 6-week-old plants were transplanted to 75 × 75 × 75 mm pots filled with peat substrate inoculated with *Berkeleyomyces* spores.

The preparation of the inoculated peat substrate was performed as follows. First, spore suspensions from each of the four isolates (OT2, OT3, WPT7, WPT8) were obtained by washing the mycelium of 6–7-day-old cultures with sterile water. Spore density in the suspension was assessed using a hemocytometer (Bürker counting chamber). Then, diluted suspensions (with density adjusted to 50 × 10^6^ spores/L) were added to the peat substrate to achieve a final density of 10,000 spores per 1 g of substrate. Inoculated peat substrate was mixed thoroughly and then distributed to small pots (approx. 85 g per pot) into which tobacco plants were transplanted, as mentioned above.

The experiment was carried out in three replicates, with six plants in each replicate inoculated with one of the four isolates. The same number of plants in each replicate constituted an uninoculated control. Shortly after transplantation into pots, each plant was supplemented with 40 mL of multicomponent mineral fertilizer ‘Kristalon zielony’ (3 g/L; Yara, Szczecin, Poland). The experimental plants were kept for four weeks in a growth chamber at 20–23 °C with a 16 h photoperiod. Subsequently, the plants were removed from the pots, their roots were washed under running tap water, and an assessment of the disease index was conducted according to the 12-grade Horsfall–Barratt scale [24]. In this scale, values of disease severity indices correspond to the ranges of the percentages of the root system affected by the disease as follows: 0: 0% (no disease symptoms), 1: 1–3%, 2: 4–6%, 3: 7–12%, 4: 13–25%, 5: 26–50%, 6: 51–75%, 7: 76–88%, 8: 89–94%, 9: 95–97%, 10: 98–99%, 11: 100% (total decay of the root system). Roots of representative plants were subjected to microscopic examination to confirm the presence of *Berkeleymocyes* sp. in tissues with disease symptoms. Subsequently, the shoots and roots of all experimental plants were dried separately at 45 °C for at least seven days, after which the dry weight of these plant parts was determined.

### 2.5. Statistical Analysis

The data collected on the morphology of fungal cultures (including culture diameter, length and width of endoconidia and chlamydospores), disease severity indices, and dry weight of roots and shoots were initially analyzed using Microsoft Excel (version 1808, Microsoft Office Standard 2019). Subsequently, the results of the pathogenicity test were subjected to analysis using Statistica version 13.3 (Tibco Software, Palo Alto, CA, USA). The effects of four different fungal isolates on the disease severity index were tested using a non-parametric Kruskal–Wallis test. Subsequently, differences among experimental groups were examined using multiple comparison tests. The effects of fungal inoculation on shoot and root dry weight were examined using analysis of variance (ANOVA). However, in cases where the assumptions of this test were not met, the Kruskal–Wallis test was employed as an alternative.

## 3. Results and Discussion

### 3.1. Molecular Identification of the Isolates

A review of sequence alignments of the tobacco isolates obtained in this study revealed that the sequences were nearly identical. Only two single nucleotide polymorphisms were detected (one in the *ACT* region and one in *MCM7* region) and they differentiated the Osiny isolates from the remaining tobacco isolates. The greenhouse-maintained isolate used in the breeding programs at IUNG-PIB was identical in the sequenced regions compared to isolates from Wola Przybysławska. Therefore, in further research, only four representative tobacco isolates were included: two from Osiny (OT2 and OT3) and two from Wola Przybysławska (WP7 and WP8). DNA sequences of these four tobacco isolates and three IPP accessions, obtained in this study, were deposited in the NCBI database under accession numbers PQ511477–PQ511483 and PQ553434–PQ553447 (Table S1). 

**Figure 2:**
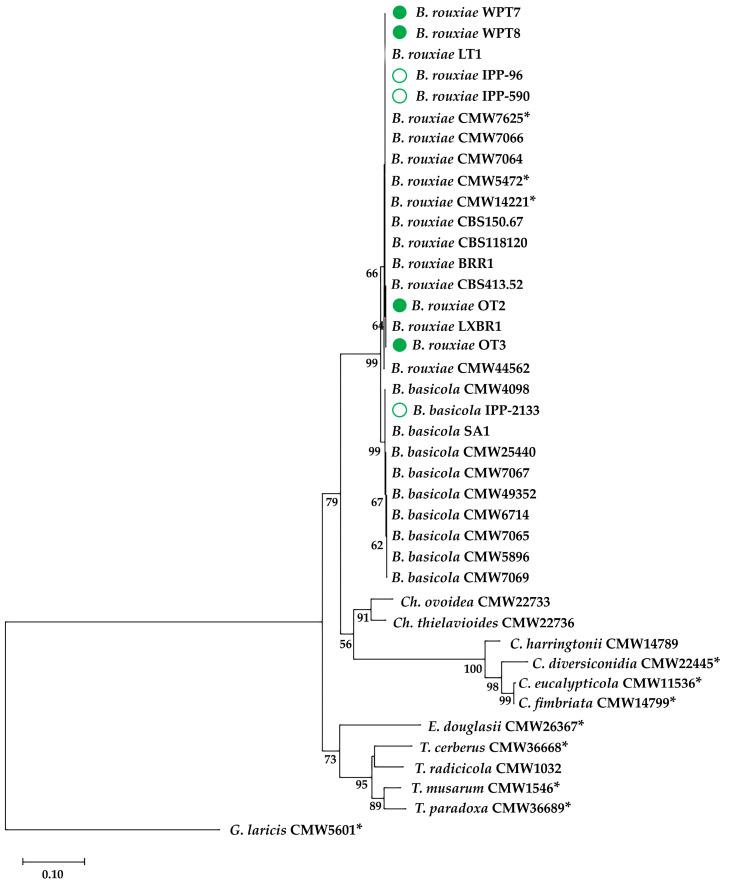
Maximum likelihood tree constructed from concatenated nucleotide sequences of ITS and *MCM7* showing relative relationship of 40 isolates of *Berkeleyomyces* spp. and other related species. Numbers below the nodes are bootstrap values of 1000 replicates (only values greater than 50% are shown). Isolates sequenced in this study are indicated in green: tobacco isolates (closed circles) and isolates obtained from the Bank of Pathogens IPP-NRI (open circles). Genus abbreviations: B—*Berkeleyomyces*, Ch—*Chalaropsis*, C—*Ceratosystis*, E—*Endoconidiophora*, T—*Thielaviopsis*, G—*Graphium*. Asterisks indicate ex-type strains.

A BLAST search of highly similar sequences in the NCBI database revealed a high (99–100%) similarity of the four tobacco isolates to *B. rouxiae* sequences deposited in this database. In the phylogenetic trees, these isolates were included in the same clusters with other *B. rouxiae* strains, including the type specimens and one tobacco strain from Switzerland (CBS150.67) sequenced by Nel et al. [8] (Figure 2 and Figure S1).

The three isolates obtained from the Bank of Pathogens (IPP-NRI, Poznań, Poland) were identified as follows: IPP-590 obtained from wheat and IPP-96 from poinsettia showed the highest sequence similarity to *B. rouxiae* accessions. On the other hand, IPP-2133 obtained from carrot represents *B. basicola*. Therefore, it is possible that both *Berkeleyomyces* species occur in Poland. Phylogenetic analyses confirmed the above-mentioned assignment of isolates to *Berkeleyomyces* spp. (Figure 2 and Figure S1).

### 3.2. Morphological Characterization of Representative Isolates

The mycelia of the four *B. rouxiae* isolates from tobacco, cultivated on PDA medium at 25 °C, exhibited moderate growth rates. Eleven days after transferring 6 mm culture discs to a fresh medium, the diameter of the colonies varied between 47.9 mm and 61.7 mm, which depended on the isolate. The isolate WP7 exhibited the highest growth rate (5.1 mm/day), followed by OT2 (4.3 mm/day) and OT3 (3.9 mm/day). The lowest growth rate was observed in the case of WPT8, at 3.8 mm/day (Table 1). Such differences may be attributed to the natural variability among the isolates. 

During the course of our studies, no notable differences in color or mycelial morphology were observed among the isolates. All colonies initially exhibited a cottony texture with a gray coloration. Subsequently, after approximately four to five days, the central part of the colony transformed into a dark-brown hue with a powdery appearance, while the margins of the colony remained light gray (cultures after 8 days of growth from the preliminary experiment are presented in Figure 1D). Earlier studies have reported that cultures of *T. basicola* may show a wide variation in morphology. In his 1950 study, Stover [25] distinguished ‘gray’ and ‘brown’ isolates on the basis of the differential morphology of pure fungal cultures. Punja and Sun [26] characterized the morphology of a large collection of 50 *T. basicola* isolates from different geographic areas and hosts, including tobacco. They identified five distinct morphological groups distinguished by cultural pigmentation that ranged from dark brown/black, gray/olive green, and light brown to white and albino. However, the tobacco isolates they examined predominantly fell within the first group, exhibiting the darkest cultures. The observations of low variation among tobacco isolates and rather dark pigmentation appear to be consistent with the findings of our study. However, Nel et al. [8] reported that the different color variants of cultures were present among isolates of both *Berkeleyomyces* species. Therefore, these differences reflect a wide intraspecific morphological variation, and the two species cannot be distinguished based on the color of the culture.

The four tobacco isolates exhibited the substantial production of endoconidia and aleuriospores. Cylindrical unicellular endoconidia were formed on elongated mycelial hyphae (Figure 1F). The average length and width of the endoconidia ranged from 13.20 to 16.53 µm and from 4.43 to 4.92 µm, respectively (Table 1). The shortest endoconidia were observed in isolate WPT7, while the longest were observed in isolate OT2. The morphology, dimensions, and cell wall structure of the endoconidia observed in this study were found to be comparable to those of *B. rouxiae* isolated from *Vicia faba* by Long et al. [13] and from *Lactuca sativa* by Nakane et al. [14]. The aleuriospores of all isolates studied were cylindrical, darkly pigmented, and consisted of one to eight chlamydospore segments (Figure 1E, Table 1). They developed laterally and terminally on the branching hyphae. Their cell walls were observed to be thick and the terminal segments were conoid. The size of the chlamydospore segments ranged from 8.05 to 8.58 µm (length) and from 10.37 to 11.71 µm (width). These data fit within the size ranges reported for *B. rouxiae* by Nel et al. [8]. The phialides were terminally formed on hyphae and exhibited a tapering shape, narrowing towards the end. Their average length ranged from 61.40 µm to 68.37 µm, recorded for isolates WPT8 and WPT7, respectively. The average width of the phialides was comparable among the four isolates (Table 1).

### 3.3. Pathogenicity Tests of the Representative Isolates

**Figure 3:**
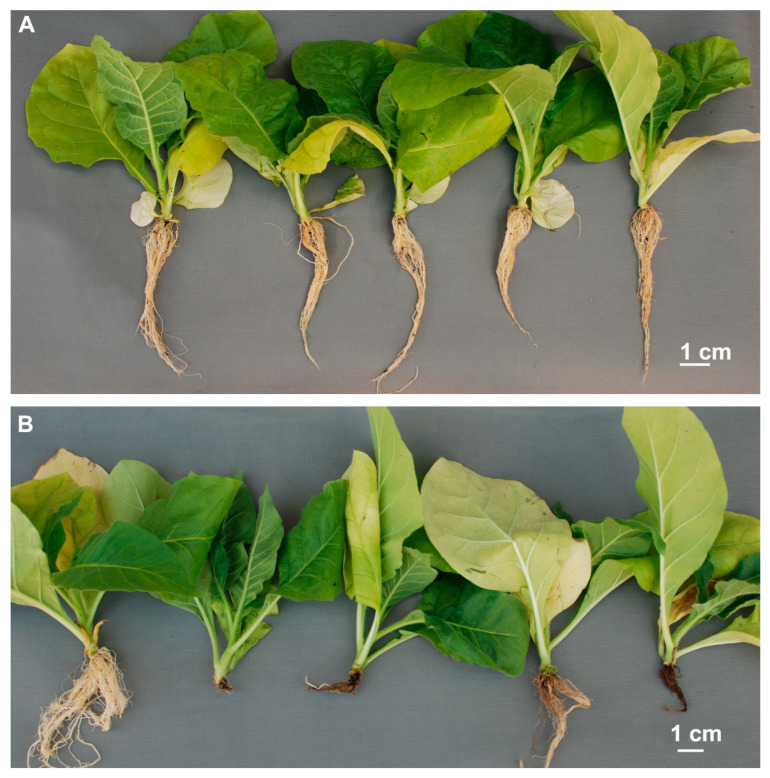
Representative plants of tobacco cultivars VRG 10TL (**A**) and Virginia Joyner (**B**) subjected to pathogenicity test with four *B. rouxiae* isolates. In both pictures, the subsequent plants represent, from the left to the right, non-inoculated control and then plants inoculated with isolates OT2, OT3, WPT7, and WPT8 (bar = 1 cm).

Four weeks after inoculation with different *B. rouxiae* isolates, plants of tobacco cv. VRG 10TL remained asymptomatic with the exception of single plants (1 out of 18 tested plants) inoculated with each of the isolates WPT7 and WPT8 (Figure 3A, Table 2). However, over all experimental VRG 10TL plants, no significant differences in the disease severity indices were detected among the inoculated plants and the control (Kruskal–Wallis test; H = 3.125, df = 4, N = 89, *p* = 0.537). Given that the median disease severity index is equal to 0 for all tested isolates, it can be assumed that the resistance of cv. VRG 10TL was effective against all four isolates. In contrast, plants of cv. Virginia Joyner inoculated with the same isolates exhibited symptoms of root rot ranging from medium to severe, with median disease severity indices between 5 and 9, depending on the isolate (Figure 3B, Table 2). Microscopic observations of the root fragments from representative plants confirmed the presence of morphological structures characteristic of *Berkeleymyces* (chlamydospores, phialides, and endoconidia) in/on the roots exhibiting disease symptoms and their absence in the asymptomatic roots. The difference in disease severity indices between the inoculated plants and the non-inoculated control of Virginia Joyner was not statistically significant only in the case of isolate WPT7 (Table 2). Inoculation with the remaining three isolates resulted in severe symptoms on plants of this cultivar (Figure 3B).

The black root rot had a deleterious effect on the growth of the susceptible cultivar Virginia Joyner. Inoculation with *B. rouxiae* isolates, which caused the most severe symptoms (OT2, OT3, WPT8), resulted also in a reduction in the dry weight of the roots and shoots compared to the control (Figure S2). The conditions of the pathogenicity tests conducted in this study closely resemble those of natural field conditions, in which young plants are planted in the soil with high pathogen pressure. Our observation that root rot affected the shoot dry weight of susceptible cultivar as early as four weeks after inoculation was not unexpected. In heavily infested fields, plants of susceptible cultivars begin to exhibit symptoms of retarded growth at an early developmental stage, before they produce a flowering stem (Figure 1A).

In contrast to the other three isolates, inoculation with the isolate WPT7 did not result in the development of severe root rot symptoms and had no significant effect on the dry weight of the shoots and roots of the inoculated plants compared to the control (Figure S2). Therefore, our pathogenicity test revealed isolate-specific differences in aggressiveness. Such differences were often observed among isolates of *T. basicola*. For example, Miczyńska and Jeziorska [27] investigated the pathogenicity of four Polish lupin- and euphorbia-isolates of *T. basicola* in tobacco cv. Virginia Joyner and demonstrated a considerable variability in aggressiveness among the isolates. Three of these isolates caused extensive root lesions, with an additional three to four weeks of post-inoculation growth inhibition. In contrast, one isolate was observed to exhibit only weak aggressiveness. The authors highlighted that the isolates under investigation showed notable morphological differences, which could be attributed to intraspecific variation. Furthermore, Stover [25] reported the occurrence of gray and brown isolates within the species and demonstrated that the gray isolate exhibited reduced aggressiveness and was more poorly adapted than the brown isolate to prolonged periods of dormancy. The findings presented in these earlier studies should be interpreted with caution due to the absence of genetic analyses that would exclude the possibility of the presence of different cryptic species among the examined isolates. In a recent study, Cavalcante et al. [9] conducted a phylogenetic analysis and pathogenicity tests on six Brazilian *Thielaviopsis*-like isolates causing black root rot on carrot. The isolates were identified as *B. basicola*. The bioassays revealed intraspecific variation in aggressiveness among the isolates, with two isolates causing severe carrot root damage, three isolates causing intermediate lesions, and one isolate causing minor root damage. In another study, four isolates obtained from cotton plants with root rot symptoms were examined [28]. A phylogenetic analysis based on the sequences of two informative gene regions confirmed the assignment of these isolates to the species *B. rouxiae*. In a pathogenicity test, two out of the four tested isolates caused the development of black lesions on the stems and significantly reduced the height and weight of cotton plants. The results of these recent studies indicate that intraspecific variation in the pathogenicity and aggressiveness of different isolates can be observed within each of the two *Berkeleyomyces* species. The availability of isolates that differ in their ability to infect the host is crucial for subsequent comparative studies aimed at identifying different pathogenicity factors.

Currently, the primary strategy for managing black root rot in tobacco crops is through the use of resistant cultivars. This approach is considered as the most efficient and environmentally friendly way to control the disease. The use of genetic sources of black root rot resistance present within the *Nicotiana* genus has been a key strategy in resistance breeding in tobacco for many years. In the early 2000s, research was conducted to exploit resistance from *Nicotiana debneyi* and *N. glauca*, resulting in the development of several cultivars resistant to black root rot [5,29,30]. Their implementation into agricultural practice has enabled the cultivation of tobacco even in regions with elevated levels of soil pathogen concentration. Nevertheless, findings from this study and a review of the literature suggest that the prolonged viability of spores of *T. basicola* and their remarkable resilience to unfavorable environmental conditions still present a significant threat to susceptible tobacco cultivars or other susceptible crops (e.g., legumes, ornamental crops).

## 4. Conclusions

This study provides a detailed characterization of the fungal isolates associated with the black root rot of tobacco in Poland. The identification of the obtained isolates was performed for the first time in the context of new taxonomy, including two *Berkeleyomyces* species. Morphological characterization assigned the isolated fungi to the *Berkelyomyces* genus. The subsequent sequencing of three informative regions allowed for the identification of these isolates as *B. rouxiae*. Four representative isolates were subjected to detailed examination of their morphology and pathogenicity test. The isolates exhibited variability in their cultural characteristics, such as growth rate and size of endoconidia and phialides. Additionally, they differed in their aggressiveness to susceptible tobacco cultivar. These isolates may serve as a valuable resource for future comparative analyses aimed at identifying different pathogenicity genes.

## Figures and Tables

**Table 1:** Morphological characterization of the four selected *Berkeleyomyces* isolates obtained from tobacco and grown on PDA medium: growth rate of the cultures and length and width of three structures (endoconida, chlamydospore segments, and phialides). For chlamydospore segments, their number per aleuriospore is also provided. The presented values are means ± standard deviations. In parentheses, the ranges (minimum–maximum) for the measurements are presented.

Isolate Name *	Culture Growth Rate (mm/Day)	Endoconidia		Chlamydospore Segments			Phialides		
		Length (µm)	Width (µm)	No. Per Aleuriospore	Length (µm)	Width (µm)	Length (µm)	Width Near Base (µm)	Width at Tip (µm)
OT2	4.3 ± 0.6	16.53 ± 1.59 (13.55–20.66)	4.51 ± 0.48 (3.18–6.04)	4.1 ± 1.2 (1–7)	8.31 ± 0.94 (5.81–10.37)	11.39 ± 0.67 (9.26–13.20)	64.98 ± 6.76 (45.82–83.54)	6.24 ± 0.84 (3.86–8.19)	4.54 ± 0.62 (2.87–6.10)
OT3	3.9 ± 0.6	16.27 ± 2.53 (11.43–25.65)	4.92 ± 0.61 (3.63–6.39)	4.3 ± 1.1 (1–6)	8.58 ± 1.31 (6.38–12.64)	11.71 ± 0.93 (9.52–15.21)	64.48 ± 7.75 (45.68–81.20)	6.28 ± 0.92 (3.87–8.43)	4.62 ± 0.65 (2.85–6.13)
WPT7	5.1 ± 0.7	13.20 ± 2.18 (8.81–19.28)	4.43 ± 0.43 (3.34–5.78)	4.4 ± 1.1 (2–8)	8.38 ± 1.20 (6.47–12.09)	10.37 ± 1.02 (7.33–12.48)	68.37 ± 9.21 (41.77–85.16)	6.32 ± 0.74 (4.14–7.85)	4.47 ± 0.60 (2.73–5.79)
WPT8	3.8 ± 0.2	15.25 ± 2.25 (11.23–23.45)	4.50 ± 0.46 (3.01–6.24)	3.9 ± 0.8 (2–5)	8.05 ± 1.00 (5.60–10.24)	11.70 ± 0.93 (9.38–13.64)	61.40 ± 5.84 (47.69–77.60)	6.32 ± 0.91 (4.31–8.66)	4.28 ± 0.64 (2.71–6.32)

* Isolates OT2 and OT3 originate from tobacco roots sampled in Osiny, while WPT7 and WPT8 originate from tobacco roots sampled in Wola Przybysławska. Both sampling sites are located in Lubelskie province (southeastern Poland).

**Table 2:** Median and range of disease severity indices recorded according to Horsfall–Barratt scale for two tobacco cultivars four weeks after inoculation with four different *B. rouxiae* isolates. Tested isolates were obtained from tobacco in two locations in southeastern Poland, in Lubelskie provice: Osiny (isolates OT2 and OT3) and Wola Przybysławska (isolates WPT7 and WPT8).

Tobacco Cultivar	Median (and Range) of the Disease Severity Indices				
	Non-Inoculated Control	OT2	OT3	WPT7	WPT8
VRG 10TL	0 (0–0)	0 (0–0)	0 (0–0)	0 (0–2)	0 (0–2)
Virginia Joyner	0 (0–0) c *	9 (3–11) a	7 (5–11) ab	5 (4–6) bc	9 (5–11) a

* Lowercase letters in the same row indicate the results of post-hoc test of multiple comparisons performed after a significant result of the Kruskal–Wallis test (H = 61.682, df = 4, N = 88, *p* = 0.000). The same letter indicates no statistically significant differences between two compared experimental groups (*p* > 0.05).

## Data Availability

Fungal cultures and DNA sequences were deposited in publicly available repositories, as stated in the manuscript. All the remaining data are contained within the article and Supplementary Materials.

## Supplementary Materials

The following supporting information can be downloaded at: https://www.mdpi.com/article/10.3390/pathogens13121120/s1, Table S1: Accession numbers of the sequences and isolates used in phylogenetic analyses. Figure S1: Maximum likelihood tree constructed from concatenated nucleotide sequences of three regions (ITS, *MCM7*, *ACT*) showing the relative relationship of 27 accessions belonging to *Berkeleyomyces* and *Ceratocysis* genera. Figure S2: Average dry weight of the shoots and roots of two tobacco cultivars subjected to testing pathogenicity of the four isolates of *B. rouxiae*.
