# Supplementary material for: Berkeleyomyces rouxiae—A Pathogen Causing the Black Root Rot of Tobacco
Source: Pathogens. 2024 Dec 18;13(12):1120. doi: 10.3390/pathogens13121120 (PMC11678428; doi:10.3390/pathogens13121120)
Supplement: Supplementary file 1 [file pathogens-13-01120-s001.zip › pathogens-3353879-supplementary.pdf]

Supplementary materials

Berkeleyomyces rouxiae—A Pathogen Causing the Black Root Rot of Tobacco

Grażyna Korbecka-Glinka *, Anna Trojak-Goluch and Diana Czarnecka

Department of Biotechnology and Plant Breeding, Institute of Soil Science and Plant Cultivation – State Research Institute, 24-100 Puławy, Poland; anngol@iung.pulawy.pl (A.T.-G.); diana.czarnecka@iung.pulawy.pl (D.C.).
* Correspondence: gkorbecka@iung.pulawy.pl

Table S1 Accession numbers of the sequences and isolates used in phylogenetic analyses. Data obtained in this study is indicated in green.

Species name	Isolate name/culture collection no.	CBS culture collection no.	ITS	MCM7	ACT
Berkeleyomyces basicola	CMW4098		MF952421	MF967078	MF967131
Berkeleyomyces basicola	CMW5896		MF952422	MF967100	MF967141
Berkeleyomyces basicola	CMW6714		MF952423	MF967079	MF967142
Berkeleyomyces basicola	CMW7065	CBS341.33	MF952424	MF967082	MF967144
Berkeleyomyces basicola	CMW7067	CBS487.48	MF952425	MF967084	MF967146
Berkeleyomyces basicola	CMW7069		MF952420	MF967085	MF967147
Berkeleyomyces basicola	CMW49352	CBS142796	MF952429	MF967102	MF967148
Berkeleyomyces basicola	CMW25440	CBS142829	MF952428	MF967088	MF967129
Berkeleyomyces basicola	SA1		MF952430	MF967108	MF967115
Berkeleyomyces basicola	IPP-2133		PQ511483	PQ553447	PQ553440
Berkeleyomyces rouxiae		CBS118120	MF952413	MF967098	MF967117
Berkeleyomyces rouxiae	CMW5472	CBS117825	MF952406	MF967080	MF967140
Berkeleyomyces rouxiae	CMW7064	CBS194.26	MF952407	MF967081	MF967143
Berkeleyomyces rouxiae	CMW7066	CBS342.33	MF952408	MF967083	MF967145
Berkeleyomyces rouxiae		CBS413.52	MF952417	MF967106	MF967120
Berkeleyomyces rouxiae		CBS150.67	MF952416	MF967107	MF967118
Berkeleyomyces rouxiae	CMW7625	CBS117828	MF952412	MF967112	MF967151
Berkeleyomyces rouxiae	CMW14221	CBS142830	MF952404	MF967113	MF967125
Berkeleyomyces rouxiae	CMW44562		MF952395	MF967089	MF967132
Berkeleyomyces rouxiae	LXBR1		ON679637	ON711036	-
Berkeleyomyces rouxiae	LT1		LC341294	LC421984	-
Berkeleyomyces rouxiae	BRR1		OK526425	OK526429	-
Berkeleyomyces rouxiae	OT2		PQ511477	PQ553441	PQ553434
Berkeleyomyces rouxiae	OT3		PQ511478	PQ553442	PQ553435
Berkeleyomyces rouxiae	WPT7		PQ511479	PQ553443	PQ553436
Berkeleyomyces rouxiae	WPT8		PQ511480	PQ553444	PQ553437
Berkeleyomyces rouxiae	IPP-590		PQ511481	PQ553445	PQ553438
Berkeleyomyces rouxiae	IPP-96		PQ511482	PQ553446	PQ553439
Ceratocystis harringtonii (syn. Ceratocystis populicola)	CMW14789	CBS119.78	EF070418	KM495435	MF967155
Ceratocystis fimbriata	CMW14799	CBS114723	KC493160	KJ601542	MF967156
Ceratocystis eucalypticola	CMW11536		FJ236723	KM495428	-
Ceratocystis diversiconidia	CMW22445	CBS123013	FJ151440	KM495423	-
Chalaropsis ovoidea	CMW22733		FJ411343	KM495487	-
Chalaropsis thielavioides	CMW22736		FJ411342	KM495489	-
Thielaviopsis cerberus	CMW36668		JX518348	KM495415	-
Thielaviopsis musarum	CMW1546		JX518325	KM495445	-
Thielaviopsis paradoxa	CMW36689		JX518342	KM495451	-
Thielaviopsis radicicola	CMW1032		KF612023	KM495459	-
Endoconidiophora douglasii	CMW26367		NR_120295	KM495424	-
Graphium laricis	CMW5601		AY148183	KM495476	-

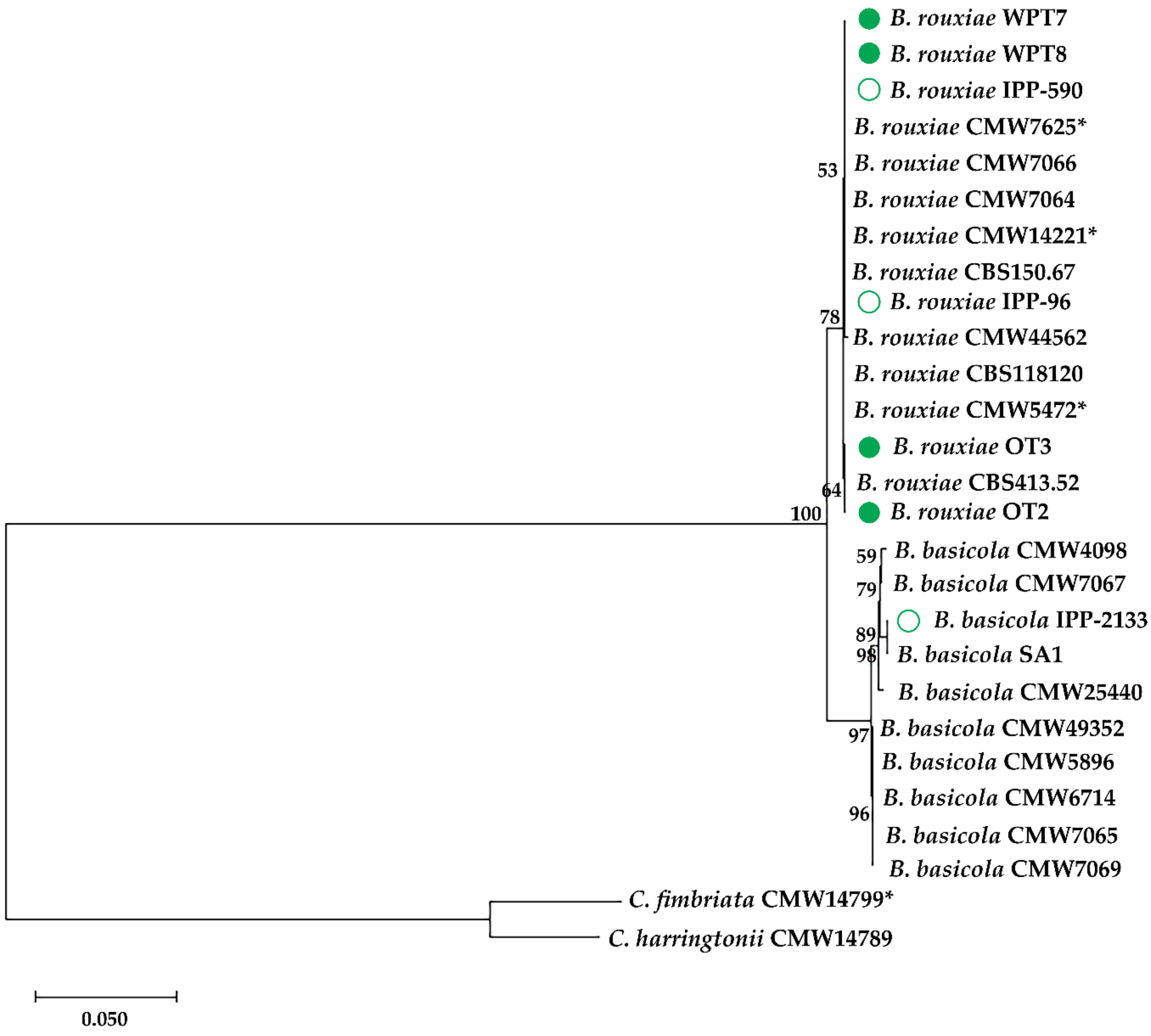

Figure S1 Maximum likelihood tree constructed from concatenated nucleotide sequences of three regions (ITS, *MCM7*, *ACT*) showing relative relationship of 27 accessions belonging to *Berkeleyomyces* and *Ceratocysis* genera. Numbers next to the nodes are bootstrap values of 1000 replicates (only values greater than 50% are shown). Isolates sequenced in this study are indicated in green: tobacco isolates (closed circles), isolates obtained from the Bank of Pathogens IPP-NRI (open circles). Asterisks indicate ex-type strains.

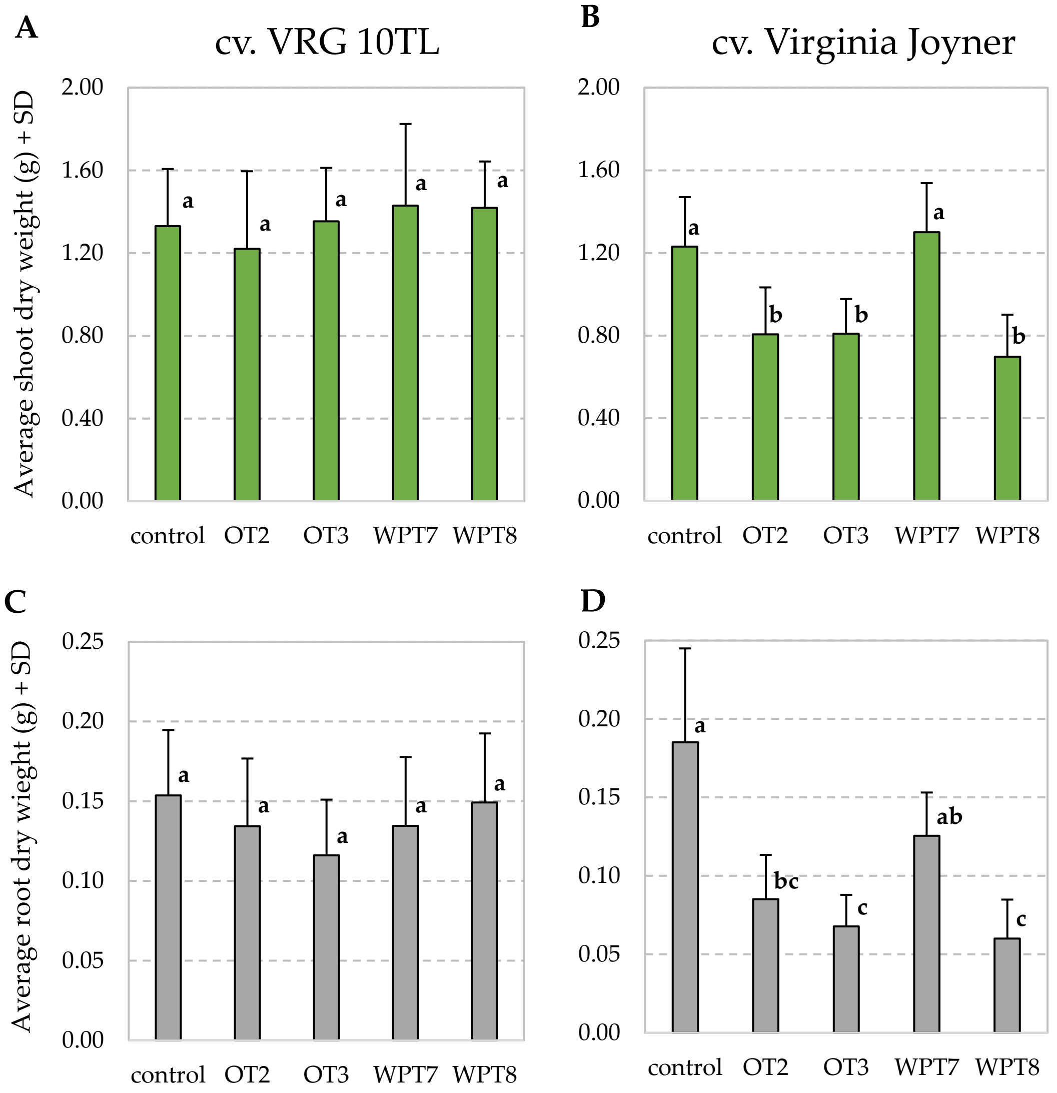

Figure S2 Average dry weight of the shoots and roots of two tobacco cultivars (VRG 10TL and Virginia Joyner) subjected to testing pathogenicity of the four isolates of *B. rouxiae* (OT2, OT3, WPT7, WPT8). The plotted values are averages + standard deviation. Lowercase letters indicate results of post-hoc tests performed after ANOVA (in case of cv. VRG 10TL; Fig. A and C) or rank ANOVA (Kruskal-Willis test in case of data for cv. Virginia Joyner; Fig. B and D). The same letter assigned for two experimental groups in the same graph indicates a lack of statistically significant differences among them.
